# Supplementary material for: Content-rich biological network constructed by mining PubMed abstracts
Source: BMC Bioinformatics. 2004 Oct 8;5:147. doi: 10.1186/1471-2105-5-147 (PMC528731; doi:10.1186/1471-2105-5-147)
Supplement: Additional File 2 — The original results of the above study (non-essential files are deleted to keep the file size under the limit set by BMC bioinformatics). [file 1471-2105-5-147-S2.bz2 › chilibotAdditionalFile2/dip10/388ID11297552E9862/html/PDGFRA_PDGFB.html]

 


 **PDGFRA** and **PDGFB** 
  
Found 113 abstracts in PubMed, retrieved 10.  
 

 What does Google say? 
 PDF only 
| .edu only 

---

**Interactive relationship** (e.g. stimulation, inhibition, etc)

**Non-interactive relationship** (e.g. studied together, co-existance, homology, etc.)

- Semi quantitativereverse transcription polymerase chain reaction was used to compare the relative abundance of 2 transcripts for housekeeping proteins beta actin and ribosomal protein L30 and 3 transcripts for growth factor ligand or receptors platelet derived growth factor  [ **PDGFB** ]  receptor alpha  [ **PDGFRA** ]  PDGFRalpha, basic fibroblast growth factor bFGF, in mature bovine oocytes of high versus low developmental potential.  Ref: 12760483 Can J Vet Res, 2003
- Characterization of the platelet derived growth factor  [ **PDGFB** ]  receptor alpha  [ **PDGFRA** ]  and c kit genes in the pufferfish Fugu rubripes.  Ref: 12592706 DNA Seq, 2002
- One of the genes was the alpha subunit of the platelet derived growth factor  [ **PDGFB** ]  receptor  **Pdgfra** .  Ref: 12605401 J Neurosci Res, 2003
- In addition, treatment with RA induced expression of the platelet derived growth factor  [ **PDGFB** ]  receptor alpha  [ **PDGFRA** ] .  Ref: 12808116 Lab Invest, 2003
- We also showed that the A549 lung cancer cells expresses the platelet derived growth factor  [ **PDGFB** ]  receptor alpha  [ **PDGFRA** ] , and the inhibitory effects of Gleevec on A549 cells is likely mediated through inhibition of PDGFR alpha phosphorylation.  Ref: 12537587 Mol Cancer< MedlineTA>Mol Cancer, 2003
- This report identifies a genetic rearrangement in the eosinophilic cell line EOL 1 that results in the expression of a fusion protein comprising an N terminal region encoded by a gene of unknown function with the GenBank accession number NM\_030917 and a C terminal region derived from the intracellular domain of the platelet derived growth factor  [ **PDGFB** ]  receptor alpha  [ **PDGFRA** ]  PDGFRalpha.  Ref: 12808148 Proc Natl Acad Sci U S A, 2003
- Although bone marrow biopsies in these patients showed increased numbers of CD25 mast cells and atypical spindle shaped mast cells, patients with HES and elevated serum tryptase could be distinguished from patients with systemic mastocytosis and eosinophilia by their clinical manifestations, the absence of mast cell aggregates, the lack of a somatic KIT mutation, and the presence of the recently described fusion of the Fip1 like 1 FIP1L1 gene to the platelet derived growth factor  [ **PDGFB** ]  receptor alpha  [ **PDGFRA** ]  gene  **PDGFRA** .  Ref: 12676775 Blood, 2003
- We found the PLATELET DERIVED GROWTH FACTOR  [ **PDGFB** ]  RECEPTOR ALPHA  [ **PDGFRA** ] , EARLY GROWTH RESPONSE PROTEIN 1 and INSULIN LIKE GROWTH FACTOR 2 genes as well as several genes associated with MYCC and ERBB2 overexpressed by at least 2 fold in metastatic tumors in both array data sets.  Ref: 12845196 Pediatr Neurosurg, 2003
